# Perceptions of Colombian Olympic coaches on talent identification and development in sport: a qualitative study

**DOI:** 10.3389/fpsyg.2026.1712976

**Published:** 2026-03-17

**Authors:** Diego Sánchez, Diogo V. Martinho, António Figueiredo, Joel Barrera-Díaz, Honorato Sousa, Nestor Ordoñez-Saavedra, Susana Ramos, Élvio R. Gouveia, Pedro Campos, Hugo Sarmento

**Affiliations:** 1 University of Applied and Environmental Sciences, Faculty of Health Sciences, Bogota, Colombia; 2 Universidade de Coimbra, Faculdade de Ciências do Desporto e Educação Física, Coimbra, Portugal; 3 Universidade de Coimbra, CIPER, Faculdade de Ciências do Desporto e Educação Física, Coimbra, Portugal; 4 CIPER, Faculdade de Motricidade Humana, Universidade de Lisboa, Cruz-Quebrada-Dafundo, Portugal; 5 Department of Physical Education and Sport, University of Madeira, Funchal, Portugal; 6 Research Center in Sports Sciences, Health Sciences, and Human Development (CIDESD), Vila Real, Portugal; 7 Interactive Technologies Institute, Laboratory of Robotics and Engineering Systems, Funchal, Portugal; 8 Department of Informatics Engineering and Interactive Media Design, University of Madeira, Funchal, Portugal; 9 WoWSystems Informática Lda, Funchal, Portugal

**Keywords:** development, expertise, identification, Olympic coaches, talent

## Abstract

**Introduction:**

Studies examining talent identification and development have primarily focused on athletic samples. However, there is limited literature addressing the determinants of talent from coaching perspectives. The aim of this study was to characterize the perspectives of coaches regarding talent identification and development among Colombian elite coaches.

**Methods:**

The research adopted a qualitative, interpretative approach, utilizing semi-structured interviews. The guide was validated through expert review and interviews conducted with 10 coaches who have Olympic experience. Thematic analysis was conducted for data analysis using NVivo software (version 14) and a deductive-inductive content analysis approach based on the ecological dynamics theoretical framework.

**Results:**

The results were organized into three main categories of constraints: (1) related to the athlete; (2) related to the task; and (3) related to environment. Participants highlighted the importance of physical, technical, tactical, and, above all, psychological skills in talent identification. Coaches also emphasized that talent should not be viewed as static but as a dynamic construct influenced by biological maturation and environmental factors. Deliberate practice and play were considered complementary in sport development, with early diversification of sporting experiences being highly valued by Olympic coaches. Family involvement, institutional support, and socioeconomic stability emerged as critical elements to ensure the continuity and sustainability of the process.

**Conclusion:**

The geographic and ethnic diversity of Colombia was recognized as a factor that can facilitate talent identification and development although structural challenges such as infrastructure shortages and the lack of coordinated national policies continue to limit the development of elite athletes. The talent development model in Colombia should consider the interrelationships between different constraints to effectively identify and develop athletes for elite-level competition.

## 1 Introduction

The identification and development of young athletes from early ages is a central focus in the field of sports. It is often described as a multifactorial process that requires coaches to have expertise in multiple areas within the sport (Abbott and Collins, 2004). The coaching capacity within the field of talent development cannot be solely focused on technical and tactical outcomes. Instead, a comprehensive coaching approach should be multidimensional, supporting the psychological and social wellbeing of athletes, while also creating a challenging environment aimed at achieving defined goals (Sherwin et al., 2017). Moreover, coaches also be aware of the importance of working with multidisciplinary teams. Apparently, experienced coaches are better able to prevent and identify changes and potential effects on performance. For this reason, they are more capable of recognizing talented athletes compared to less experienced coaches (Roberts et al., 2021).

The process of identifying and developing talent in sport can be viewed as a ‘temporal continuum’ consisting of two phases: (1) identifying and selecting athletes for immediate performance, and (2) identifying and selecting athletes based on potential future performance (predicting performance) (Roberts et al., 2019). Throughout this continuum, the environment created for athlete development is crucial, with coaches playing a central role. They are responsible for organizing and managing the key factors that influence the development of young athletes (Xiang et al., 2024). Therefore, challenging environments should be designed to provide optimal conditions for youth to reach their maximum potential (Sargent Megicks et al., 2022). As previously mentioned, talent identification and development are multifactorial, non-linear processes, which some authors have described as atypical (Roberts et al., 2019). In fact, research has focused on developing predictive models for athlete performance, attempting to identify variables that are decisive for adult success (Den Hartigh et al., 2018; Romann et al., 2024). However, some of these studies did not account for the context and evolutionary trends of sport, which limits the accuracy of the models. Performance prediction is complex, and therefore, coach experience and decision-making remain central to meeting athletic needs (Cushion et al., 2012; Pankhurst et al., 2013). Most literature on talent in sports has focused exclusively on athletes’ experiences, performance, and perceptions of the factors that determine their attainment of elite status (Güllich, 2018, 2019; Sarmento et al., 2026). Moreover, research on talent pathways has primarily focused on athletes from Europe and North America, particularly the United States, with few studies conducted in South America (Barth et al., 2024; Sanchéz et al., 2025). In contrast, there is a scarcity of studies examining coaches’ comprehension of talent identification and development. This gap is surprising, given that coaches play a crucial role in making critical decisions that influence athletes’ pathways to expertise in their respective sports. A previous study conducted with 16 coaches in Britain evaluated the talent development system in the UK (Martindale et al., 2007). This research helped identify the difficulties within the system and highlighted strategies for improvement.

Coaches are currently seeking the most effective protocols to identify talented athletes—those associated with the lowest error rates in talent assessment (Denison et al., 2019; Werneck et al., 2024). Meanwhile, the coaches’ perception of talented athletes has not aligned with scientific evidence, raising questions about the reliability of their subjective judgment in selecting athletes with future potential (Cripps et al., 2019). Another central issue for coaches is creating the appropriate environment to nurture the potential talent of athletes (Sanchéz et al., 2025). The lack of studies involving South American samples represents a significant gap in the literature. Colombia, in particular, is characterized by its rich cultural, ethnic, and geographic diversity, which provides unique conditions for the development of youth athletes (World Bank, 2020). However, social and economic constraints serve as barriers for those athletes who have the potential to reach an elite level (Lemaitre Ripoll et al., 2014). With this in mind, the aim of this study was to explore Olympic Colombian coaches’ regarding multidimensional factors in the identification and development of elite athletes. Investigating the perceptions of Olympic coaches regarding talent identification and development could yield new insights into the field of sports talent development.

## 2 Methods

### 2.1 Study design

The study was grounded in the physiological-scientific principle of the interpretative paradigm, taking into account the ontological and epistemological understandings of reality that are influenced by interactions and individual subjectivities. Based on this framework, our aim was to understand the phenomena within contextual and cultural scenarios, which are dependent on the perspectives of the interviewees (Lincoln and Eudond, 1991; Mertens and Ginsberg, 2009). Qualitative research offers unique insights into the motivations, values, and needs of coaches in the field of talent identification and development. By adopting the interpretative paradigm, we assume that the perception and consciousness of coaches can be understood and analyzed in a continuous way, since the context is constantly evolving in a dynamic manner. Ontological relativism suggests that there is no single or objective reality, but rather a subjective reality shaped by the beliefs and contextual interactions of coaches (Creswell, 2015). Epistemological constructivism suggests that knowledge is dynamically shaped through social interactions and interpretations (Bryman, 2016). These philosophical perspectives underscore the importance of a holistic and contextual understanding of the lived experiences of coaches working with Olympic athletes in relation to talent identification and development.

### 2.2 Participants

The study involved 10 male Colombian coaches (mean age 46.2 ± 8.7 years) with experience in soccer, swimming, and athletics (21.1 ± 7.2 years of experience). All coaches held a master’s degree in Sports Science. As an inclusion criterion, only coaches who had participated in at least one Olympic Games were considered for this study. Participants provided written informed consent and participated voluntarily and anonymously.

### 2.3 Instrument

In the present study a semi-structured interview was performed. The interview guidance was validated through a review process, which involved five specialists. These experts were selected using a technique called individual aggregation. All the specialists were informed about the purpose and evaluation process of the interview and participated independently in reviewing the guidance (Barrera-Díaz et al., 2023). The experts were selected based on the following criteria: (1) three specialists held PhDs in sports science with a focus on quantitative data; (2) two specialists were coaches actively involved in talent selection processes. The interview guidance was organized into four main dimensions related to talent identification and development: (1) the conceptualization of talent; (2) constraints associated with the task; (3) constraints related to the athlete; and (4) constraints related to the environment.

### 2.4 Procedure and analysis

The video call interview was conducted synchronously using the Microsoft Teams platform, lasting a minimum of 37 min and a maximum of 52 min. The duration of the interview depended on the comfort level experienced by the interviewees concerning the topics discussed and the extent of self-reflection each considered necessary (Polkinghorne, 1989). The interviews were conducted by the first author (DS) between November 2024 and April 2025, and the audio recordings were used for analysis. Subsequently, to perform the qualitative content analysis, the interviews were transcript for a Word document (Microsoft Corporation, Redmond, WA, USA) and the information was codified using a deductive-inductive approach. The transcripts were read repeatedly to promote familiarity with the data and immersion in the underlying content (Creswell and Poth, 2016; Sousa et al., 2024). By reading the interviews repeatedly, the first author (DS) observed significant reflections on the statements made by the coaches, allowing for the initiation of a reflective process and engagement with their perspectives. After becoming familiar with the interview content, the main themes and potential relationships were identified. These themes were then interpreted collaboratively with the rest of the research team and considered within the adopted theoretical framework. The data were analyzed using the inductive analysis process in three steps, following the methodology outlined by Thomas and Harden (2008). Initially, each interview transcript was coded by unit of meaning, and these codes were then organized into descriptive themes. These themes were later analyzed in relation to the research questions, providing a synthesized interpretation of the data. Afterwards, the themes were refined (merged or separated) to offer a more comprehensive understanding of the emerging concepts. Taking into account the ecological dynamics theoretical framework, talent is shaped by the dynamic relationships among various constraints, including the environment, task, and player (Davids et al., 2017). Talent should not be considered an innate trait; instead, it emerges as a functional relationship between the athlete and these constraints (Davids et al., 2013). This approach has been discussed in previous studies (Sarmento et al., 2018; Mendes et al., 2025) and was used to organize the results section.

The codification of interviews was performed using the NVivo software (version 14; Lumivero) due to the thematic analysis (Smith, 2016). This study was conducted under Resolution 8.430 of 1993 of the Colombian Ministry of Health, classified as minimal risk research, and approved by the Ethics Committee of the Faculty of Health Sciences, Sports Science Program at the University of Applied and Environmental Sciences (Acta - CD-1-2024).

### 2.5 Methodological rigor

The current study followed the recommendations of Creswell (2009) and Creswell and Miller (2000) to ensure the validity of qualitative research. These steps included: (1) the first author verifying the transcriptions to ensure accuracy and eliminate errors; (2) the first author coordinating communication among the research team through regular meetings; and (3) two authors independently coding the transcriptions to achieve reproducibility in the coding process. Member verification is an important step to ensure data quality in qualitative research. In this study, the results were shared with the coaches to confirm the accuracy and validity of the interpretations. This process occurred at two stages: (1) at the end of each interview, allowing coaches to review and amend their responses; and (2) the transcriptions were emailed to each coach for review and final approval. At this stage, participants could add, delete, or modify their statements. Additionally, coaches were invited to contact the research author if they wished to make further changes to their statements.

## 3 Results

**Figure 1:**
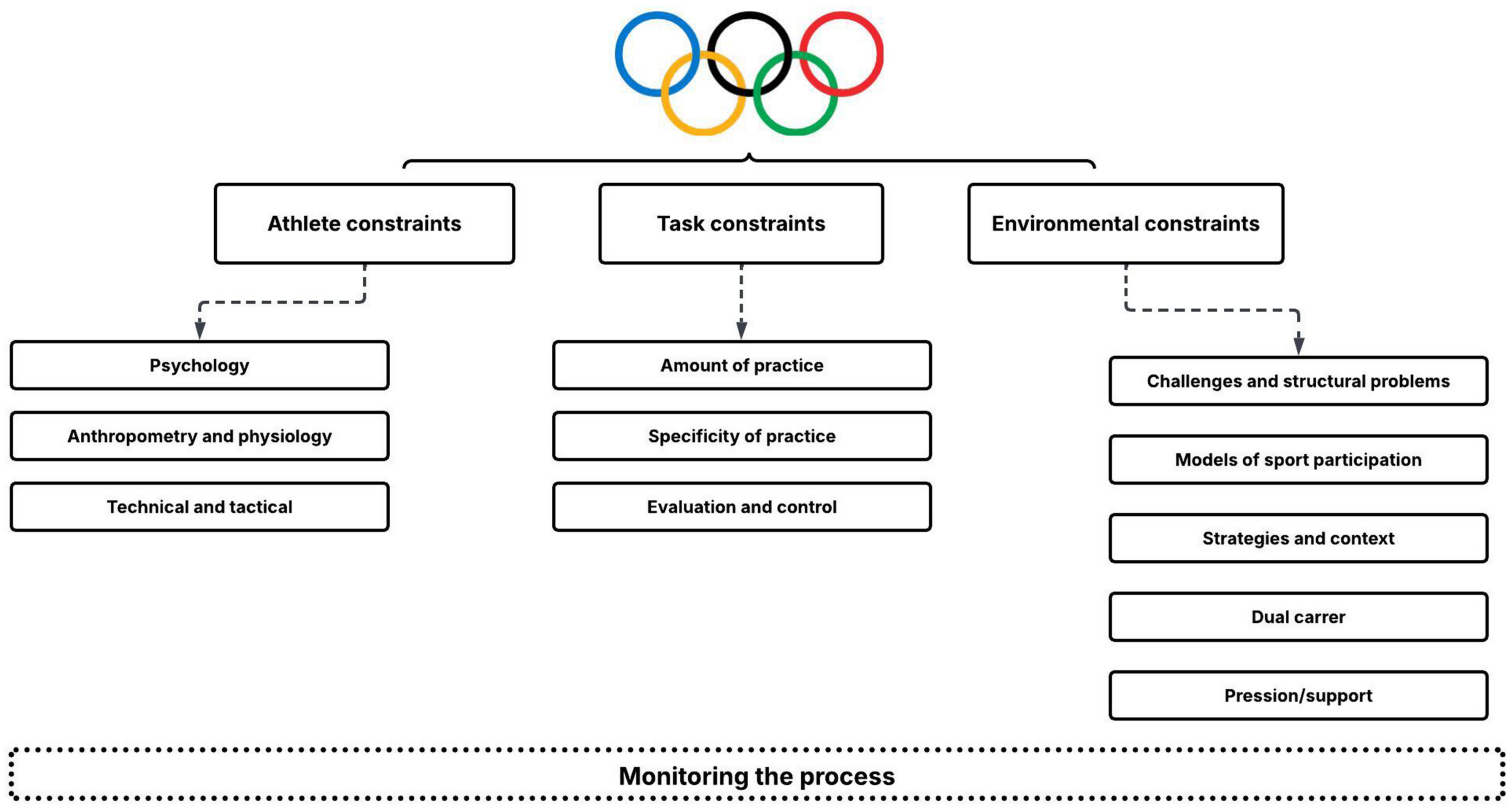
Constraints associated with the talent identification and development process.

Based on the ecological-dynamic framework (Figure 1), which assumes that human behavior is understood through the dynamic relationship between the participant and the environment—affected by personal constraints, tasks, and context—the presentation of results was organized into three main categories: (1) athlete constraints; (2) task constraints; and (3) environmental constraints (Davids et al., 2013; Davids et al., 2017).

### 3.1 Athlete constraints

The interviewed coaches, when attempting to define what they understand by a talented athlete, generally agreed on a general perspective of an individual who stands out in sports practice. This distinction can be based either on skills related to expertise in a specific sport or on physical and/or psychological aspects. Regarding the key elements for talent selection some coaches consider game intelligence and technical skills as the primary indicators, while others believe that physical attributes are decisive to define talent. They also highlight that the characteristics of different sports influence the processes of identifying and selecting potential talented athletes.

Talent in sport can be defined as a person who is in a state of above-average development, characterized by physical, psychological, and physiological attributes. These particular characteristics that confer an advantage to a given athlete may enable them to develop high-level skills and performance capacity in their specific sport.—Coach 1

The fundamental elements are identifying skills related to physical and technical dimensions, followed by a psychological assessment, whether in training or competition contexts. How they behave in these two settings is, in my view, essential.—Coach 2

It always depends on the context and the sport in question, but for me, the component of game intelligence, combined with precise technical execution, is the most important element. Of course, those who are faster, stronger, or more coordinated tend to stand out, but greater attention should be given to those who demonstrate the ability to manage their actions within the game space according to the objective and show intentionality in their actions.—Coach 10

When coaches mentioned the specificity of different sport modalities, there was a consensus about the criteria used to identify talented players in team sports whilst, in individual sports the physical capacities have more influence on explaining talent. The advancement in biological maturation is often considered to identify talent in individual sports. Nevertheless, there is a widespread view that coaches should be attentive and guide practitioners toward other sports environments due to characteristics that may enhance participation in different types of sports.

I believe that this is more apparent and easier to recognize in team sports, which even facilitates migration between sports, especially when the development process begins in another modality with shared active game principles. In individual sports, however, it does not seem to be as straightforward—for example, starting practice based on an individual physical and technical skills development process, and later transitioning to another sport with a different framework or approach.—Coach 2

The analysis of the results showed that coaches did not consistently rank the importance of specific characteristics for performance. However, some interviewed coaches considered game intelligence and psychological components to be the most important attributes. The interviews also highlighted that physical capacities, whether at the initial stages or in advanced phases of sports practice, are crucial for achieving expertise in sport.

Regarding the likelihood of achieving significant performance at a high level, for me, the most important factor is the psychological aspect, above technical and physical components. These should ideally be developed equally, but always under the influence of psychological mastery.—Coach 4

I find it very difficult to establish an order of importance among the factors influencing this process, as each plays a specific role and has particular influence. Viewing the human being holistically, I believe this approach makes assessment imprecise. What exists are developmental factors that enhance participation in certain sports, particularly physical characteristics resulting from genetics. Perhaps, in some cases, there's a greater tendency to prioritize this dimension initially. In such situations, technical and psychosocial components are usually more emphasized during training processes rather than during the initial assessment and talent identification, especially in certain sports.—Coach 5

### 3.2 Task constraints

The time dedicated to deliberate practice and the intentionality behind it were identified as decisive factors for reaching the elite level. Coaches highlighted that, beyond the amount of practice, the organization and structuring of developmental processes are more important. The quantity of practice was primarily considered a decisive factor in endurance sports.

Currently, I am convinced that 10,000 hours of deliberate practice are not necessary. Concerning specialization and sporting success, I would be much more concerned with the quality and structuring of practice rather than the total accumulated hours throughout the athlete’s developmental trajectory. It is also important to consider the age group of the practitioners. In my opinion, it is crucial to accumulate hours of deliberate practice between the ages of 6 or 7 and 12 or 13, but not exclusively in the main sport. They should also gain experience in other sports and always engage in recreational athletic activities that foster the development of different skills. After 12 or 13 years of age, specialization based on deliberate practice in the specific sport should begin. Until that age, in my view, it makes more sense for practitioners to participate in controlled processes that allow them to experience a variety of contexts and activities, which increase adaptability and facilitate the transfer of motor skills to enhance the specialization phase in the specific sport.—Coach 2

Cyclical sports (i.e. endurance sports) require many hours of development and, consequently, significantly more hours of deliberate practice. However, it is more important to have coherence and efficiency in planning the total number of hours of deliberate practice than to simply accumulate 10,000 hours in a practice that does not foster motor adherence, sensitivity, or sport-specific knowledge.—Coach 5

On the other hand, play-based practice can foster creativity, which some closed training methodologies do not permit. Some coaches also mentioned the use of play-form activities within training sessions, controlled by coaches, to develop more autonomous and adaptable practices.

Free practice is important because it lacks the control and rigidity found in many training methodologies. It provides the athlete with 100% freedom of choice, something that no coach or structured training program can fully reproduce. Additionally, it involves different stimuli, a variety of information, and personal interpretation. The athlete experiments, absorbs the experience, interprets it, and develops their own understanding. As a result, athletes become more empowered, more creative, and more autonomous. There is less extreme dependence on the coach, as is often the case in organized training processes.—Coach 1

It is through play-based games during early childhood that creativity and motor development are enhanced, largely due to free and continuous play activities that foster natural motivation in children and require diverse body control. Nowadays, there is much discussion about the need to incorporate such moments and activities into the regular training planning. However, the real issue is that this need is often only discussed because these activities have likely disappeared from children’s lives in the so-called street context.—Coach 7

Regarding the control and evaluation of training, two main ideas emerged: (1) coaches are not able to reconcile the training process with the need to regularly assess the capacities of athletes. Coaches assume that the organization and planning of training and competitions are conducted on a daily basis; (2) coaches also noted that they use the assessment of different capacities as a benchmark to monitor the development of youth athletes from childhood through adulthood.

The focus tends to be more on the daily planning and management of exercise and training, often neglecting the monitoring of athletic talent progression. The process heavily relies on individuals' ability to assess and evaluate.—Coach 4

It should be perceived as a long-term process, beginning with an initiation phase for children aged 6 to 12, where evaluation and monitoring should concentrate on technical skills and their development. Around ages 12 to 16, greater attention is given to physical development, marked by observable physical characteristics and indicators of biological maturation, whether advanced or not. From 16 to 18 years, the focus shifts toward sporting performance, with an emphasis on achieving results, and where competition acts as a key indicator for controlling and assessing development.—Coach 7

Coaches recognized the importance of monitoring and assessing athletes’ capacities for proper training periodization and, consequently, athletic development. Furthermore, clubs need to incorporate specialized staff in various domains. If this is not possible, establishing protocols with academic institutions is essential to assess the capacities of athletes.

It is essential for clubs to have qualified human resources, even if they are university interns. This group can enhance the quality of evaluations, including those beyond obvious physical assessments. I am referring to tests focused on cognitive and social evaluations, which provide valuable additional information to the process—something unlikely to occur if resources are only allocated to on-field training, due to limited time and specialized knowledge.—Coach 3

As a coach, it is quite difficult to be responsible for training planning, evaluation, and maintaining objectivity in that assessment, as it is inherently part of our work and recognizing potential errors can be challenging. Additionally, finding a coach with specialized expertise to evaluate all domains is often problematic. Typically, we establish protocols in collaboration with universities.—Coach 7

The evaluation of the training process helps to determine whether the training methods are appropriate for a specific stage of athlete development and also enables adjustments to be made based on sport performance.

Evaluation and monitoring help ensure that the development plan is aligned with the athlete’s goals, whether in terms of physiological adaptations or managing training load volumes and intensities. It allows for adjustments in case of shortcomings or poor performance and the preservation and documentation of effective practices, which can serve as indicators for future progress — all within a framework focused on the athlete’s ongoing, progressive development.—Coach 3

### 3.3 Environmental constraints

The Colombian context is characterized by cultural, ethnic, historical, and geographic diversity, which imparts unique characteristics that require more in-depth analysis concerning the development of elite athletes. Several challenges and structural weaknesses were identified, including a lack of qualified human resources and infrastructure, as well as limited support from the educational system for those aspiring to become elite athletes.

The primary challenges mainly stem from the absence of suitable infrastructure for certain sports and a shortage of well-qualified human resources. Economic inequalities limit not only the acquisition of sports equipment but also discourage many children from engaging in sports. Another common issue is the instability within sports organizations, characterized by frequent coach turnover. Since coaches are poorly paid, there is a greater need to find individuals willing to accept these low salaries. Consequently, people without professional qualifications may end up performing coaching duties, which can affect the quality and consistency of training—Coach 2.

I believe that one significant challenge—both problematic and demanding—is to finally bridge the gap between the sports sector and the educational system. This is crucial to support elite athletes in pursuing their academic development alongside their sporting careers, as well as to leverage the expertise of the human resources trained within academia. Such integration would not only enhance the quality of training and talent development but also promote greater rigor in the oversight and management of these processes. Without this closer connection, we risk continuing with inadequate systems incapable of meeting the demands of international competition—Coach 6.

Coaches reported three different perspectives about the impact of social and cultural level of the country in the development of athletic potential: (1) low socioeconomic and cultural level; (2) the second perspective highlights how structured environments promoting the athletic development; (3) the context of social hardship can potential specific traits such as increased resilience for training and competition, along with a possible drive to achieve higher levels of performance.

Socioeconomic factors significantly influence young athletes, affecting the purchase of personal sports equipment in the early stages of training, as well as transportation to training sessions and competitions. These challenges often lead to early dropout, precisely when young athletes are still developing their sporting skills and acquiring important social values and principles.—Coach 3

It is easier to boost athletic performance when the environment surrounding the young athlete is stable.—Coach 4

The social and cultural background clearly influences young athletes. A strong spirit of achievement, transcendence, and ambition is deeply rooted in those coming from more disadvantaged social environments. Additionally, there is a recognition of the symbolic value these athletes hold for society, should they succeed in reaching high levels of professional achievement.—Coach 8

On the other hand, socioeconomic difficulties also drive the development of recruitment strategies, such as interventions with families to explain the development process. The provision of sports scholarships appears to be widely accepted, as they not only enhance training conditions but also offer convenience and foster greater psychosocial stability for athletes.

An effective communication strategy involves holding introductory briefings about the clubs’ sports projects for the athletes' families. Support and maintaining close ties with the family are fundamental. Additionally, a more technical approach should explain the long-term development and training plan, including the prioritized training contents, volume and intensity of workouts, the types of tournaments to participate in, and the follow-up and assessment methods used to monitor the athlete’s progress.—Coach 3

One of the most frequently used strategies is the sports scholarship, which eliminates any costs for the young athlete during their talent development. Another widespread approach is bringing the athlete to the club’s facilities, providing adequate conditions for nutrition, accommodation, and schooling.—Coach 4

There are specific geographic zones in Colombia associated with the development of talented athletes in certain sports. Coaches also noted that regions with lower levels of social and infrastructural development have fewer opportunities to identify talented athletes compared to cities located in coastal areas. Rural towns are considered important for sports that do not require extensive material or physical resources. Additionally, cities with a higher prevalence of Afro-descendant athletes are viewed as excellent areas for talent identification, particularly considering physical attributes.

Colombia has several regions well-suited for identifying talented athletes and areas with a strong sporting culture. Antioquia, Atlántico, and the Chocó region are known for their strong football traditions, producing many international players who have represented the national team. However, the potential for sporting talent is widespread throughout Colombia, regardless of the region.—Coach 2

In discussing the most appropriate environments for talent identification, schools and their extracurricular sports activities were highly valued, along with youth tournaments that have a long tradition in the country. Despite concerns about safety in some regions, which are considered too dangerous for play-practice, particularly on the streets, coaches believe that specific areas continue to serve as talent pools, with high participation rates driven by socioeconomic disadvantages.

In the early stages, school sports and extracurricular activities enable children to explore and develop their full potential, offering valuable early insights into those with more advanced motor skills. Additionally, sport academies operate within age-specific limits. Recognized tournaments centered on the more competitive aspect of each sport serve as platforms where more specialized and developed athletes begin to emerge. Ultimately, well-structured youth categories within clubs can attract children from a very young age, provided there is strong organization at that level.—Coach 10

All coaches also highlighted the crucial role of schools in facilitating dual careers, which positively influences long-term athlete development and wellbeing. However, they also expressed some dissatisfaction with the support provided by the educational and academic systems for the sports careers of young athletes.

The educational development of young athletes is essential. In my view, the key issue lies in the education system’s failure to adapt to talented athletes at the highest levels. There is a lack of coherence between the school workload, curriculum content, and the need to accommodate athletes who, for example, represent the country and cannot attend classes daily. While the importance of academic education in sports is acknowledged, the academic sector still appears to underestimate the value of sports practice itself—Coach 3.

In my view, the main differentiator is that clubs are better structured, with more qualified human resources who understand the advantages of supporting a dual career, assisting young athletes in managing their time and academic responsibilities. This success hinges on clubs investing more in qualified personnel and enhancing their structures with individuals who can add value across all domains of young athlete development.—Coach 5

The idea of a dual career is essential for the long-term well-being of the athlete. It enables emotional growth and a greater sense of peace with oneself, as it goes beyond just training and competition, providing the opportunity to develop in other areas as well.—Coach 7

In this regard, the role of the coach is central to the athlete’s development, as they often serve as the primary educator and role model for young athletes, given the significant amount of time spent together. Therefore, building trust and fostering effective communication are essential. It is also important to recognize that the relationship between coach, athlete, and family should never be solely focused on athletic performance.

The ideal is for the relationship to be highly positive and based on mutual trust, given the unique nature of sport… The coach also plays an educational role. Effective communication is essential, particularly for the coach to fully understand the broader context outside of the club.—Coach 8

The relationship should not be purely sports-focused, as there are elements of a young athlete’s development that are not directly tied to performance or results, and in which the coach can assist the family, and vice versa. Furthermore, information shared within the home environment may be relevant to training, and there are situations that the young athlete may only communicate to their coach. Consequently, it is crucial to maintain high levels of trust and ensure effective communication channels to optimize the athlete's routine and development.—Coach 9

In Colombia, there is a tendency to adopt successful practices and models from other countries. However, the sociocultural context and available resources in Colombia differ from those in European countries. This disparity should be considered in the talent identification and development programs implemented in Colombia.

Every country has its own distinct characteristics, whether related to collective social norms or individual differences among its inhabitants. Effective intervention requires a thorough assessment of the specific context, identifying elements with the potential for positive influence, and adapting the model with tailored interventions. Transferring a foreign model in its entirety is often too complex, particularly when the countries involved have markedly different attributes.—Coach 1

While having references is valuable, the most appropriate approach always involves a comprehensive development model that integrates physical, technical, tactical, and mental aspects. Additionally, the effectiveness of training largely depends on the coach’s competence in managing the training process. Nowadays, there is significant transfer of information from the European context to South America. The important thing is to identify the positive elements of these European models—whether in training programs or structural reforms in sports policy—and adapt them to our cultural realities and available resources.—Coach 2

## 4 Discussion

The involvement of sports coaches in the talent identification and development process is crucial for long-term athletic success. The investigation of talent in the context of sports is challenging due to the significant variation in its definition from coach to coach. In this study, some coaches refer to talent as an extraordinary athlete based on overall performance, while others focus on specific abilities. The phenome of talent in sport is dynamic and affected by the different constraints. Accordingly, this study aimed to explore the perceptions of Colombian Olympic coaches regarding these processes, drawing on their extensive experience working with elite athletes. The findings were organized into three main categories of constraints: (1) related to the athlete; (2) related to the task; and (3) related to environment.

Developing a structured process for talent selection and development is a key priority for clubs, federations, and Olympic organizations (Johnston et al., 2018). Analyzing the conceptual understanding of the interviewed coaches regarding the concept of talent and the factors that enhance athletic development, we can conclude that specific theoretical and practical frameworks are applicable across different sports. In this context, performance prediction emerges as a difficult and challenging process for coaches. Studies highlighted that prediction of performance are often based on the coach’s subjective insights and perceptions of an athlete’s potential (Güllich and Cobley, 2017). Coaches are often relied upon to validate the results of tests used in talent identification and development programs. Consequently, the success of these methods heavily depends on the accuracy of the predictions made by coaches, despite limited scientific evidence confirming the validity of such assessments (Roberts et al., 2019). The coaches interviewed generally define talented athletes as those who stand out above their peers. To define talent, coaches focus on visible characteristics and immediate performance, especially in the physical, biological, technical, and psychological domains (Larkin and O'Connor, 2017; Milistetd et al., 2013). However, coaches recognized the challenge of making early judgments, as athletic development is affected by maturation and environmental factors. Nonetheless, there is considerable pressure to accelerate the development of early-maturing athletes to achieve short-term competitive results, which may lead to neglecting other important variables that influence long-term success (Güllich et al., 2022).

The coaches interviewed highlighted the critical role of psychological factors in the development of high-performance athletes. While the importance of these factors in sports performance is widely recognized, research specifically examining their role among Colombian athletes is lacking. However, existing scientific evidence from diverse contexts around the world indicates that athletes at the highest competitive levels tend to exhibit strong goal commitment, resilience, self-regulation, problem-focused coping strategies, and the capacity to perform under pressure (Dohme et al., 2019; Gledhill et al., 2017; Sarmento et al., 2018). In Colombia, understanding and developing these traits is especially important, given the social and environmental challenges many athletes face. From an ecological perspective, these skills should not be seen as fixed, internal traits but as adaptable, socially mediated capacities shaped by the practice environment (Sarmento et al., 2018). Consequently, programs that incorporate psychological training within sport-specific contexts could play a crucial role in enhancing performance. The Psychological Characteristics of Developing Excellence questionnaire is a valuable tool for those working with talented athletes, as it measures six psychological and behavioral categories that significantly influence their development. These categories include long-term success, imagery during practice and competition, coping with performance pressure, engagement in quality practice, evaluation of performance and weaknesses, and support from others to help athletes compete at their full potential (Macnamara and Collins, 2013). This questionnaire effectively differentiates a considerable percentage of athletes categorized as very good or good from those classified as very poor or poor, based on coaches’ evaluations (Macnamara and Collins, 2013). Given that coaches in the current study emphasize the importance of psychological aspects in developing youth athletes, incorporating these instruments into a holistic approach to talent development is essential.

Technical and tactical skills were also acknowledged by the coaches as essential domains for talent identification and development. Evidence shows that highest competitive athletes tend to show better technical abilities and tactical competence across various sports. In addition, anthropometric and physiological factors significantly influence sporting success, though they should be interpreted with caution. Most research indicates that elite athletes are generally taller, leaner, and demonstrate higher speed, agility, coordination, and aerobic endurance. However, individual differences in biological maturation can affect these variables (Han et al., 2023; Papai et al., 2025; Silvino et al., 2024). Given that, in the Colombian context, the development pathways for youth athletes are unequal, prioritizing early identification based on body size, maturation, or performance is not advisable. Instead, coaches should concentrate on creating an appropriate environment that fosters the development of young athletes. Meanwhile, there remains uncertainty about how to structure training environments that effectively address the specific challenges of modern sport. The ecological dynamics approach can aid coaches in creating more representative practice environments by adjusting the constraints and demands of the sport, thereby facilitating athletes’ tactical and technical adaptation.

The amount and characteristics of practice have been extensively studied in the context of sports expertise (Coutinho et al., 2016; Güllich and Emrich, 2014; Sarmento and Araújo, 2021), which likely explains why coaches consider these factors essential in talent development. Studies have investigated the impact of deliberate practice and deliberate-play as fundamental activities for learning. Deliberate practice involves structured, demanding tasks specifically designed to improve performance, typically conducted under expert supervision. It is an intentional, cognitively challenging form of training, more frequent of the later stages of athlete development (Erickson et al., 1993; Erickson et al., 2007; Erickson et al., 2008;). However, deliberate practice has faced increasing criticism by undervaluing the influence of individual and contextual factors (e.g., biological maturation, social support, access to resources, and sociocultural conditions) which are especially noted in Colombia. Conversely, deliberate play refers to informal, enjoyable, and intrinsically motivating activities that do not directly target performance but foster creativity, adaptability, and early engagement with play forms. Play practice allows athletes to develop a broad perceptual-motor foundation that supports more effective specialization in later adolescence. Both deliberate practice and deliberate play contribute synergistically to the development of sporting expertise. It is common for high-performance athletes to have accumulated substantial amounts of both types of activities throughout their careers (Coutinho et al., 2016; Hornig et al., 2016; Sarmento et al., 2018). Considering the unequal access to sport, economic instability and lack of training facilities, many Colombian athletes have developed their talent in informal environments, where play practice has played a dominant role. In this regard, it is essential to understand sports development as a non-linear process, where different pathways or trajectories—shaped by ecological, cultural, and social factors—converge toward excellence.

The constraints associated with athletic environment influence the interpretation of the results in the current study. In this context, it is important to recognize that the present sample is situated within a unique cultural, social, geographical, and historical context that deeply shapes the experiences of its inhabitants in general, and its athletes in particular. On one hand, the rugged geography and high altitude influence physiological capacities, on the other hand social resilience, develop over decades of historical instability, potentiate psychological competitive traits. In summary, understanding the sport success in Colombia involve an integrated perspective of these factors. The various models of sports participation emphasize the types of activities that athletes should engage in from infancy to adulthood. For example, the model proposed by Côté et al. (2007) consists of three distinct phases: the sampling years (ages 6–12), the specializing years (ages 13–15), and the investment years (age 16 and older). During these phases, coaches should focus on different types of practices tailored to the developmental stage of each athlete. Additionally, the Long-Term Athlete Development Model proposed by Balyi and Hamilton (2004) highlights the significance of age at peak height velocity in defining training protocols. While developmental models for youth athletes primarily focus on task constraints, elite coaches believe that other constraints, such as individual, environmental, and social factors are crucial for reaching elite levels of performance. Therefore, it is essential for models of long-term sport participation to include characteristics specific to an athlete’s age, access to appropriate equipment and facilities, and to emphasize the importance of social values. These findings align with the conclusions of a scoping review that highlights the theoretical and methodological frameworks used to investigate talent development models (Hauser et al., 2022). The study emphasizes the impact of both sport and non-sport environments, as well as the importance of various stakeholders in the development of young athletes. The origin of elite athletes in Colombia appears to be associated with specific geographic regions within the country. Certain areas seem to facilitate the development of particular sports. However, geographical location alone does not seem to be the sole determining factor, as talent development results from a complex interplay of socioeconomic, cultural, infrastructural, and other factors that influence access to sports opportunities and, consequently, the processes of nurturing talent (Hancock et al., 2022). Populations in the Colombian Pacific and Western regions, known as the birthplaces of many talented athletes, are also characterized by low population densities. This aligns with the findings of Côté et al. (2006), which indicate that athletes born in smaller cities (with populations under 500,000) are more likely to become professional players compared to those born in larger cities (with populations over 500,000). This study utilized a convenient sample of Olympic coaches to describe their perceptions of talent identification and development. Future research should focus on understanding the attainment of elite levels in Colombia by inter-relating data from athletes, environment, and training practices.

## 5 Conclusion

The results of this study indicate that the process of sports talent identification and development is closely linked to the sociocultural, ecological, and institutional complexity within which athletic models are designed. Coaches provided a critical and comprehensive perspective, highlighting that talent in sport does not emerge solely from a single factor (e.g., physical or technical attributes), but rather from the dynamic interaction of psychological skills, diverse practice experiences, and supportive environments. There are valid and useful tools for those who lead with talented athletes, allowing them to drive and monitor evidence-informed practices. Understanding talent as a multidimensional and adaptive phenomenon underscores the need for flexible pedagogical approaches that consider the specificities and needs of athletes, while also taking into account geographic, structural, and historical conditions. Finally, the results highlight the crucial role of the coach as a strategic mediator among the various stakeholders involved in talent development. The coach emerges as an agent of transformation, responsible for creating sustainable and adequate practice environments. However, the effectiveness of this mediation depends on ongoing training and institutional recognition, practices that have recently been enhanced within the Colombian context, exemplified by the recent approval of the Colombian Coaches’ Law.

## Data Availability

The raw data supporting the conclusions of this article will be made available by the authors without undue reservation.

## References

[ref1] Abbott A. Collins D. (2004). Eliminating the dichotomy between theory and practice in talent identification and development: considering the role of psychology. J. Sports Sci. 22, 395–408. doi: 10.1080/02640410410001675324, 15160593

[ref2] Balyi I. Hamilton A. (2004). Long-term athlete development: trainability in childhood and adolescence. Olympic Coach 16, 4–9.

[ref3] Barrera-Díaz J. Figueiredo A. J. Field A. Ferreira B. Querido S. M. Silva J. R. . (2023). Contemporary practices of physical trainers in professional soccer: a qualitative study. Front. Psychol. 14:1101958. doi: 10.3389/fpsyg.2023.1101958, 37799523 PMC10548828

[ref4] Barth M. Güllich A. Macnamara B. N. Hambrick D. Z. (2024). Quantifying the extent to which junior performance predicts senior performance in Olympic sports: a systematic review and meta-analysis. Sports Med. 54, 95–104. doi: 10.1007/s40279-023-01906-0, 37676619 PMC10799111

[ref5] Bryman A. (2016). Social Research Methods. Oxford: Oxford University Press.

[ref6] Côté J. Baker J. Abernethy B. (2007). “Practice and play in the development of sport expertise,” in Handbook of Sport Psychology. eds. Eklund R. Tenenbaum G. (Hoboken, NJ: Wiley), 184–202.

[ref7] Côté J. Macdonald D. J. Baker J. Abernethy B. (2006). When "where" is more important than "when": birthplace and birthdate effects on the achievement of sporting expertise. J. Sports Sci. 24, 1065–1073. doi: 10.1080/02640410500432490, 17115521

[ref8] Coutinho P. Mesquita I. Fonseca A. M. (2016). Talent development in sport: a critical review of pathways to expert performance. Int. J. Sports Sci. Coach. 11, 279–293. doi: 10.1177/1747954116637499

[ref9005] Creswell J. W. (2009). Research design: Qualitative, quantitative, and mixed methods approaches (3rd ed.). Thousand Oaks, CA: Sage.

[ref9001] Creswell J. W. (2015). Educational research: Planning, conducting, and evaluating quantitative and qualitative research (5th ed.). Pearson.

[ref9006] Creswell J. W. Miller D. L. (2000). Determining validity in qualitative inquiry.Theory Pract. 39, 124–130. doi: 10.1207/S15430421TIP3903_2

[ref9] Creswell J. W. Poth C. N. (2016). Qualitative Inquiry and Research Design: Choosing among Five Approaches. Thousand Oaks, CA: Sage Publications.

[ref10] Cripps A. J. Hopper L. S. Joyce C. (2019). Can coaches predict long-term career attainment outcomes in adolescent athletes? Int. J. Sports Sci. Coach. 14, 324–328. doi: 10.1177/1747954119848418

[ref11] Cushion C. Ford P. R. Williams A. M. (2012). Coach behaviours and practice structures in youth soccer: implications for talent development. J. Sports Sci. 30, 1631–1641. doi: 10.1080/02640414.2012.721930, 23016800

[ref12] Davids K. Araújo D. Vilar L. Renshaw I. Pinder R. (2013). An ecological dynamics approach to skill acquisition: implications for development of talent in sport. Talent Dev. Excell. 5, 21–34.

[ref13] Davids K. Gullich A. Shuttleworth R. Araújo D. (2017). “Understanding environmental and task constraints on talent development,” in Routledge Handbook of Talent Identification and Development in Sport. eds. Baker J. Cobley S. Schorer J. Wattie N. (London: Routledge), 192–206.

[ref14] Den Hartigh R. J. Hill Y. Van Geert P. L. (2018). The development of talent in sports: a dynamic network approach. Complexity 2018:9280154. doi: 10.1155/2018/9280154

[ref15] Denison J. Jones L. Mills J. P. (2019). Becoming a good enough coach. Sports Coach. Rev. 8, 1–6. doi: 10.1080/21640629.2018.1435361

[ref16] Dohme L. C. Piggott D. Backhouse S. Morgan G. (2019). Psychological skills and characteristics facilitative of youth athletes' development: a systematic review. Sport Psychol. 33, 261–275. doi: 10.1123/tsp.2018-0014

[ref17] Erickson K. Bruner M. W. MacDonald D. J. Côté J. (2008). Gaining insight into actual and preferred sources of coaching knowledge. Int. J. Sports Sci. Coach. 3, 527–538. doi: 10.1260/174795408787186468

[ref18] Erickson K. Côté J. Frasser-Thomas J. (2007). Sport experiences, milestones, and educational activities associated with high-performance coaches' development. Sport Psychol. 21, 302–316. doi: 10.1123/tsp.21.3.302

[ref19] Erickson A. Krampe R. Tesch-Römer C. (1993). The role of deliberate pratice in the acquisition of expert performance. Psychol. Rev. 100, 273–305.

[ref20] Gledhill A. Harwood C. Forsdyke D. (2017). Psychosocial factors associated with talent development in football: a systematic review. Psychol. Sport Exerc. 31, 93–112. doi: 10.1016/j.psychsport.2017.04.002

[ref9004] Güllich A. (2019). Macro-structure of developmental participation histories and micro-structure of practice of German female world-class and national-class football players. J. Sports Sci. 37, 1347–1355. doi: 10.1080/02640414.2018.1558744 30582400

[ref9003] Güllich A. (2018). Sport-specific and non-specific practice of strong and weak responders in junior and senior elite athletics - A matched-pairs analysis. J. Sports Sci. 36, 2256–2264. doi: 10.1080/02640414.2018.1449089 29519195

[ref21] Güllich A. Cobley S. (2017). “On the efficacy of talent identification and talent development programmes,” in Routledge Handbook of Talent Identification and Development in Sport. (London: Routledge), 80–98.

[ref22] Güllich A. Emrich E. (2014). Considering long-term sustainability in the development of world class success. Eur. J. Sport Sci. 14, S383–S397. doi: 10.1080/17461391.2012.706320 24444233

[ref23] Güllich A. Macnamara B. N. Hambrick D. Z. (2022). What makes a champion? Early multidisciplinary practice, not early specialization, predicts world-class performance. Perspect. Psychol. Sci. 17, 6–29. doi: 10.1177/1745691620974772 34260336

[ref24] Han M. Gómez-Ruano M. A. Calvo A. L. Calvo J. L. (2023). Basketball talent identification: a systematic review and meta-analysis of the anthropometric, physiological and physical performance factors. Front Sports Act. Living 5:1264872. doi: 10.3389/fspor.2023.1264872, 38033652 PMC10686286

[ref25] Hancock D. J. Vierimaa M. Newman A. (2022). The geography of talent development. Front. Sports Act. Living 4:1031227. doi: 10.3389/fspor.2022.1031227, 36275442 PMC9582327

[ref26] Hornig M. Aust F. Güllich A. (2016). Practice and play in the development of German top-level professional football players. Eur. J. Sport Sci. 16, 96–105. doi: 10.1080/17461391.2014.982204, 25440296

[ref9007] Hauser L.-L. Harwood C. Höner O. OConnor D. Wachsmuth S. (2022). Talent development environments within sports: A scoping review examining functional and dysfunctional environmental features. Int. Rev. Sport Exerc. Psychol. 17, 1105–1131. doi: 10.1080/1750984X.2022.2129423

[ref27] Johnston K. Wattie N. Schorer J. Baker J. (2018). Talent identification in sport: a systematic review. Sports Med. 48, 97–109. doi: 10.1007/s40279-017-0803-2, 29082463

[ref28] Larkin P. O'Connor D. (2017). Talent identification and recruitment in youth soccer: recruiter's perceptions of the key attributes for player recruitment. PLoS One 12:e0175716. doi: 10.1371/journal.pone.0175716, 28419175 PMC5395184

[ref29] Lemaitre Ripoll J. García Jaramillo S. Ramírez Rodríguez H. (2014). Vivienda/violencia: intersecciones de la vivienda y la violencia intrafamiliar en Ciudad Bolívar, Bogotá. Rev. Estud. Soc. 50, 71–86.

[ref30] Lincoln Y. Eudond G. (1991). Naturalistic Inquiry. New York: Sage.

[ref31] Macnamara A. Collins D. (2013). Do mental skills make champions? Examining the discriminant function of the psychological characteristics of developing excellence questionnaire. J. Sports Sci. 31, 736–744. doi: 10.1080/02640414.2012.747692, 23194087

[ref32] Martindale R. J. Collins D. Abraham A. (2007). Effective talent development: the elite coach perspective in UK sport. J. Appl. Sport Psychol. 19, 187–206. doi: 10.1080/10413200701188944

[ref33] Mendes D. Martinho D. V. Travassos B. Gouveia É. R. Saavedra N. O. Sarmento H. (2025). Talent selection in Portuguese national futsal teams. J. Hum. Kinet. 99, 253–261. doi: 10.5114/jhk/199379, 41245960 PMC12612822

[ref34] Mertens D. Ginsberg P. (2009). The Handbook of Social Research Ethics. Thousand Oaks, CA: Sage Publications.

[ref35] Milistetd M. Mesquita I. Sobrinho A. S. Carrara P. Nascimento J. (2013). Coaches representation about detection and selection of talents on the Brazilian volleyball. Int. J. Sports Sci. 3, 157–162. doi: 10.5923/j.sports.20130305.03

[ref36] Pankhurst A. Collins D. Macnamara Á. (2013). Talent development: linking the stakeholders to the process. J. Sports Sci. 31, 370–380. doi: 10.1080/02640414.2012.733821, 23088326

[ref37] Papai Z. Szakaly Z. Wilhelm M. (2025). Selection criteria in the talent identification of triathlon. Physiol. Int. 112, 118–137. doi: 10.1556/2060.2025.00519, 40424065

[ref38] Polkinghorne D. E. (1989). “Phenomenological research methods,” in Existential-Phenomenological Perspectives in Psychology: Exploring the Breadth of Human Experience. (Heidelberg: Springer), 41–60.

[ref39] Roberts A. H. Greenwood D. A. Stanley M. Humberstone C. Iredale F. Raynor A. (2019). Coach knowledge in talent identification: a systematic review and meta-synthesis. J. Sci. Med. Sport 22, 1163–1172. doi: 10.1016/j.jsams.2019.05.008, 31133481

[ref40] Roberts A. H. Greenwood D. Stanley M. Humberstone C. Iredale F. Raynor A. (2021). Understanding the "gut instinct" of expert coaches during talent identification. J. Sports Sci. 39, 359–367. doi: 10.1080/02640414.2020.1823083, 32962508

[ref41] Romann M. Javet M. Hernandez J. Heyer L. Trösch S. Cobley S. . (2024). Longitudinal performance trajectories of young female sprint runners: a new tool to predict performance progression. Front. Sports Act. Living 6:1491064. doi: 10.3389/fspor.2024.1491064, 39763484 PMC11700731

[ref42] Sanchéz D. Martinho D. V. Araújo D. Gouveia É. R. Figueiredo A. Saavedra N. . (2025). Training and competition pathways to the Olympic games: insights from developmental experiences of Colombian Olympians. Int. J. Sports Sci. Coach. 1–10. doi: 10.1177/17479541251363874

[ref43] Sargent Megicks B. Till K. Rongen F. Cowburn I. Gledhill A. Mitchell T. . (2022). Examining European talent development environments: athlete, parent and coach perceptions. J. Sports Sci. 40, 2533–2543. doi: 10.1080/02640414.2023.2172800, 36724148

[ref44] Sarmento H. Anguera M. T. Pereira A. Araújo D. (2018). Talent identification and development in male football: a systematic review. Sports Med. 48, 907–931. doi: 10.1007/s40279-017-0851-7, 29299878

[ref45] Sarmento H. Araújo D. (2021). A geração de ouro - Viagem ao processo que revolucionou o futebol português. Lisboa: Lisbon Press.

[ref9002] Sarmento H. Martinho D. Pereira F. Pereira A. Araújo D. (2026). The road to expertise in U-20 football world champions: A multi-method approach. Int J. Sports Sci. Coach.17479541261420672.

[ref46] Sherwin I. Campbell M. J. Macintyre T. E. (2017). Talent development of high performance coaches in team sports in Ireland. Eur. J. Sport Sci. 17, 271–278. doi: 10.1080/17461391.2016.1227378, 27598851

[ref47] Silvino V. O. Ferreira C. P. Figueiredo P. Prado L. S. Couto B. P. Pussieldi G. A. . (2024). Variables used for talent identification and development in soccer: a scoping review. Kinesiology 56, 268–280. doi: 10.26582/k.56.2.9

[ref48] Smith B. (2016). “Narrative analysis in sport and exercise: how can it be done?” in Routledge Handbook of Qualitative Research in Sport and Exercise. (Abingdon, Oxon, UK: Routledge), 282–295.

[ref49] Sousa H. Sarmento H. Gouveia É. R. Clemente F. M. (2024). Perception of professional Portuguese soccer players about the replacement of leadership with the season underway – a qualitative study. Int. J. Perform. Anal. Sport 25, 653–674. doi: 10.1080/24748668.2024.2436802

[ref50] Thomas J. Harden A. (2008). Methods for the thematic synthesis of qualitative research in systematic reviews. BMC Med. Res. Methodol. 8:45. doi: 10.1186/1471-2288-8-45, 18616818 PMC2478656

[ref51] Werneck F. Z. Coelho E. F. Matta M. O. Silva R. C. P. Figueiredo A. J. B. (2024). Goldfit soccer: a multidimensional model for talent identification of young soccer players. Res. Q. Exerc. Sport 95, 895–909. doi: 10.1080/02701367.2024.2347983, 38885196

[ref52] World Bank (2020). Poverty & equity brief. Colombia. Available online at: https://databank.worldbank.org/data/download/poverty/33EF03BB-9722-4AE2-ABC7-AA2972D68AFE/Global_POVEQ_COL.pdf (Accessed January 08, 2026).

[ref53] Xiang C. Dong W. Kamalden T. F. T. Ismail N. Luo H. (2024). Structural analysis of environmental factors of sports talent development. Curr. Psychol. 43, 6516–6532. doi: 10.1007/s12144-023-04803-x

